# Causal relationship between immune cells and risk of heart failure: evidence from a Mendelian randomization study

**DOI:** 10.3389/fcvm.2024.1473905

**Published:** 2025-01-23

**Authors:** Wenjing Cao, Zefu Yang, Liumei Mo, Zhenhao Liu, Jiawei Wang, Zhenhong Zhang, Kui Wang, Wei Pan

**Affiliations:** ^1^ Department of Cardiology, Department of Geriatric Medicine, Foshan Women and Children's Hospital, Foshan, Guangdong, China; ^2^ Department of Cardiology, The Sixth Affiliated Hospital, School of Medicine, South China University of Technology, Foshan, Guangdong, China; ^3^ Department of Cardiovascular Medicine, Pingxiang People's Hospital, Jiangxi, China; ^4^ Department of Critical Care Medicine, Jieyang Third People's Hospital, Jieyang, Guangdong, China; ^5^ Department of Cardiology Medical, The Second People's Hospital of Foshan, Foshan, China; ^6^ The First Clinical Medical College, Qilu Hospital of Shandong University, Jinan, Shandong, China

**Keywords:** causal inference, MR analysis, immunity, heart failure, genome-wide association study

## Abstract

**Background:**

Heart failure (HF) is a clinical syndrome resulting from structural damage or dysfunction of the heart. Previous investigations have highlighted the critical involvement of immune cells in the progression of heart failure, with distinct roles attributed to different types of immune cells. The objective of the current research was to explore the potential connections between immune characteristics and the development of HF, as well as to ascertain the nature of the causality between these factors.

**Methods:**

To assess the causal association of immunological profiles with HF based on publicly available genome-wide studies, we employed a two-sample Mendelian randomization technique, utilizing the inverse variance weighted (IVW) method as our primary analytical approach. In addition, we assessed heterogeneity and cross-sectional pleiotropy through sensitivity analyses.

**Results:**

A two-sample Mendelian randomization (MR) analysis was conducted using IVW as the primary method. At a significance level of 0.001, we identified 40 immunophenotypes that have a significant causal relationship with HF. There is a significant causal relationship between these phenotypes and heart failure. These immunophenotypes, 8 of which were in B cells, 5 in cDC, 2 in T cell maturation stage, 2 in monocytes, 3 in myeloid cells, 7 in TBNK and 13 in Treg. Sensitivity analyses were conducted to validate the strength and reliability of the MR findings.

**Conclusions:**

Our study suggests that there appears to be a causal effect between multiple immune cells on heart failure. This discovery provides a new avenue for the development of therapeutic treatments for HF and a new target for drug development.

## 1 Introduction

Heart failure is a clinical syndrome resulting from either structural abnormalities or dysfunction of the heart. It is a serious condition associated with significant morbidity and mortality and represents the terminal phase of the cardiovascular disease continuum, which includes conditions such as coronary artery disease and hypertension (1). Global Burden of Disease (GBD) studies have shown that there are currently approximately 68 million people with heart failure worldwide and the prevalence is increasing, representing a huge burden to society (2).

Cardiac inflammation is the major pathophysiological mechanism of heart failure. Numerous studies have implicated immune cells and inflammatory molecules in the onset and escalation of a range of cardiovascular diseases (3, 4). Immune cells, along with their secreted cytokines, are crucial in the cardiac inflammatory process and have a significant impact on promoting fibrosis and oxidative stress (4). Inflammation in the early stages of heart failure protects the host by removing inflammatory ligands, mediating apoptosis and phagocytosing necrotic tissue. Nonetheless, if inflammation persists and there is an imbalance between pro-inflammatory and anti-inflammatory mediators, it can result in detrimental remodeling of the cardiac architecture, interstitial fibrosis, and a decrease in myocardial contractile function. These pathological alterations can progress to left ventricular dysfunction and ultimately exacerbate into full-blown heart failure (1, 5).

Currently, drugs used to treat cardiac remodelling include RAAS inhibitors, beta-blockers, SGLT2 inhibitors and aldosterone receptor antagonists. These drugs usually aim to modify haemodynamics, such as controlling blood pressure, reducing afterload and blood volume. However, current mortality rates are as high as 21.6%–36.5% for acute heart failure and 6.0%–15.6% for chronic HF. Innovative therapeutic options are essential to curtail the incidence of HF, enhance patient prognoses, and alleviate the financial strain heart failure imposes on healthcare systems (2). The study suggests that the inflammatory and fibrotic processes in the heart may be reversible in both experimental models and clinical practice. This discovery opens up the possibility of immunotherapy as a promising new treatment approach and paves the way for the identification and development of fresh therapeutic targets (4, 6). Clinical trials have attempted non-specific immunomodulatory treatments, including selective inhibition of TNFα, immunosorption, intravenous immunoglobulin, plasma exchange, etc., but there is a lack of large clinical trials with little success (7, 8).

MR is a statistical technique grounded in the principles of Mendel's laws of inheritance (9–12). Using genetic variation as instrumental variables, this technique is designed to assess the causal relationship between between exposure variables and disease endpointsand, with the goal of offering a scientific foundation for the observed correlation (13–16). Given that genetic variations are randomly assigned at conception, MR can eliminate confounding factors and reverse causation bias compared to traditional methods. Previous research has uncovered various correlations between immune cell characteristics and HF, and these findings provide a theoretical basis for the association between the two. In conducting this investigation, we implemented a two-sample MR analysis to elucidate the causal connection between immune cell features and HF. Our study contributes to the discovery of the distinct regulatory roles of immune cell subpopulations in HF, thereby guiding the development of new immunotherapies to address this growing public health problem.

## 2 Materials and methods

### 2.1 Study design

**Figure 1:**
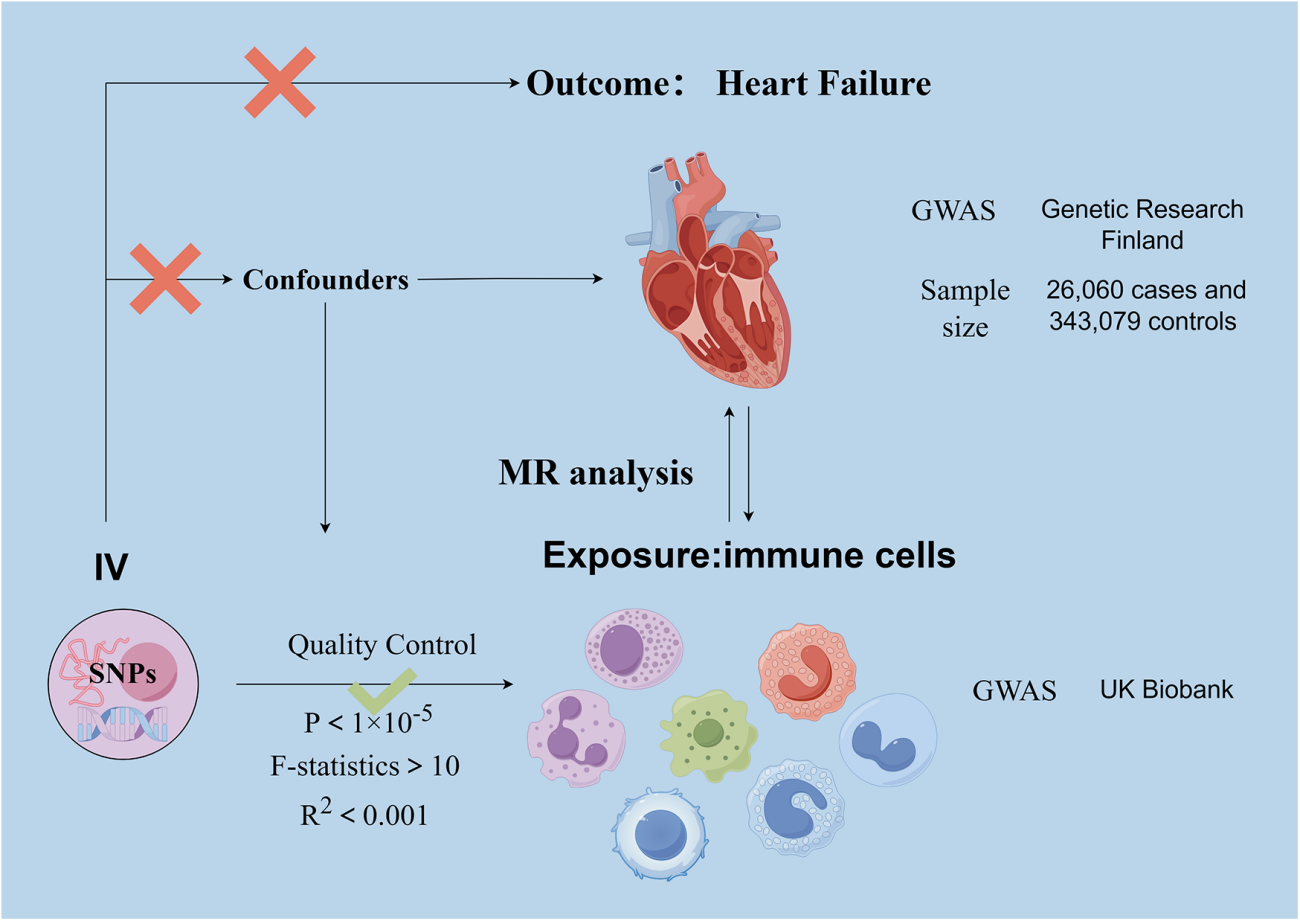
Basic principle and assumptions of Mendelian randomization analyses. Assumption 1, significantly associated with exposure; Assumption 2, not associated with outcome; Assumption 3, not associated with confounders. SNPs, single-nucleotide polymorphisms; MR, Mendelian randomization.

Our investigation delved into the causal relationships between 731 immune cell traits and heart failure using two-sample MR analyses. MR studies utilize genetic variants, known as instrumental variables (IVs), which are closely linked to certain exposure risk factors. This method allows researchers to deduce the causal impacts between these exposure variables and disease outcomes. This approach aids in mitigating the impacts of confounding factors and the issue of reverse causation, thereby improving the accuracy of causal inferences about exposure-outcome associations. Due to the random assignment of genetic variants at the time of conception and remain stable throughout life, their use as instrumental variables is effective in controlling for the problems of confounding and reverse causation that are common in observational studies. This makes MR a powerful tool for assessing causal relationships between disease risk factors and outcomes. The exposure and outcome samples in this study were human subjects and all datasets used in this study are in the public domain; it is a secondary analysis of published data and therefore does not require ethical approval. Figure 1 shows the overall design of the MR analysis, which was applied to evaluate the risk of HF associated with 731 immune cell traits.

### 2.2 Genome-wide association study (GWAS) data sources for HF

HF genome-wide association study GWAS pooled data from Genetic Research Finland, published in December 2023, available at https://finngen.gitbook.io/documentation. FinnGen is a large-scale genomics project that analyses more than 500,000 samples from the Finnish Biobank and conducts correlation studies between genetic variants and health status data. The aim of the project is to delve deeper into the mechanisms and genetic predispositions of a wide range of diseases. Driven by Finnish research institutions, biobanks and international industry partners, the FinnGen project aims to build a robust database of gene-disease associations to facilitate the development of new drugs and the practice of personalised medicine. The study performed a GWAS on 118,870 European individuals (Ncase = 26,060, Ncontrol = 343,079).

### 2.3 Immunity-wide GWAS data sources

Statistical summaries of GWAS for each immune trait are usually publicly available through the GWAS Catalogue. Data on genetic variations in relation to immune cells can be searched by visiting the website https://gwas.mrcieu.ac.uk/datasets/ and using the appropriate login numbers. The information you have provided has login numbers ranging from GCST90001391 to GCST90002121, which correspond to specific datasets and can be used to find and download data. The GWAS of Immune Cell Traits used data from 3,757 individuals of European ancestry, ensuring that there was no overlap between these data sets. Potential confounders such as sex and age were corrected to accurately assess the association between immune cell traits and genetic variants. With this approach, we aimed to identify genetic markers associated with immune traits, providing a basis for further exploration of the biological role of these traits and their potential impact on disease development.

### 2.4 Selection of instrumental variables (IVs)

In causal inference studies, valid IVs must meet the following three core assumptions: (1) there should be a direct correlation between the genetic variation and the exposure of interest (in this case, the circulating immune cell trait); (2) the genetic variation should not be correlated with other confounders that may affect the exposure-outcome relationship; and (3) the genetic variation should not have an effect on the outcome in a pathway other than through the exposure (i.e., no other confounding pathways). These presuppositions are essential for guaranteeing that the instrumental variables correctly mirror the causal link between the exposure and the outcome. Significant *P*-values for each immune cell trait for instrumental variables were established at 1 × 10^−5^, utilizing the R software (version 4.2.2) for the genome-wide analysis. The significance level for the instrumental variables (IVs) associated with each immunological trait was set at 1 × 10^−5^. To ensure unbiased results, we applied a chain imbalance threshold of 5,000 kb and an R^2^ value of less than 0.001 to the chain imbalance distance. To reduce the risk of bias caused by weak instrumental variables (IVs), we measured the strength of association with exposure factors by calculating the F-statistic for each IV. Those IVs with an F-statistic greater than 10 were considered to have a stronger ability to predict HF and were therefore more likely to be effective in detecting a causal relationship between exposure and HF.

### 2.5 Statistical analysis

In this study, we used a variety of MR analysis methods, including inverse variance weighting (IVW), MR Egger, weighted median, MR-PRESSO and simple weighting model. For the core analyses, which assessed the causal relationship between 731 immune cell characteristics and the risk of HF, we mainly used the IVW method. The relative change in HF risk per one standard deviation (SD) increase in risk factor levels was quantified by odds ratio (OR). In this study, we used the Cochrane Q statistic to test for heterogeneity between instrumental variables, and if the *P*-value was greater than 0.05, it was considered that there was no significant heterogeneity between instrumental variables. To test for the presence of horizontal pleiotropy, we performed an MR Egger regression analysis, and if the *P*-value obtained was greater than 0.05, we considered that there was no evidence of horizontal pleiotropy. In addition, to assess the effect of individual single nucleotide polymorphisms (SNPs) on overall MR estimates, we performed a leave-one-out analysis. Furthermore, scatter plots were utilized to demonstrate that outliers did not impact the outcomes.

## 3 Results

### 3.1 An overview of IVs

After a genome-wide significance threshold screen (*P* < 1 × 10^−5^) and F-statistical validation, multiple SNPs were identified as IVs among 731 immune cell SNPs. For the retained SNPs, the F-statistics were all greater than 10 (Supplementary Table S1), indicating their suitability as strong instruments. We performed MR-Egger intercept test and MR-PRESSO global test to exclude SNPs with pleiotropy (*P* < 0.05 for MR-PRESSO global test, *P* < 0.05 for MR-Egger regression). After Cochran's *Q*-test, SNPs with heterogeneity (*P* < 0.05) were excluded.

### 3.2 Exploration of the causal effect of immunophenotypes on HF

**Figure 2:**
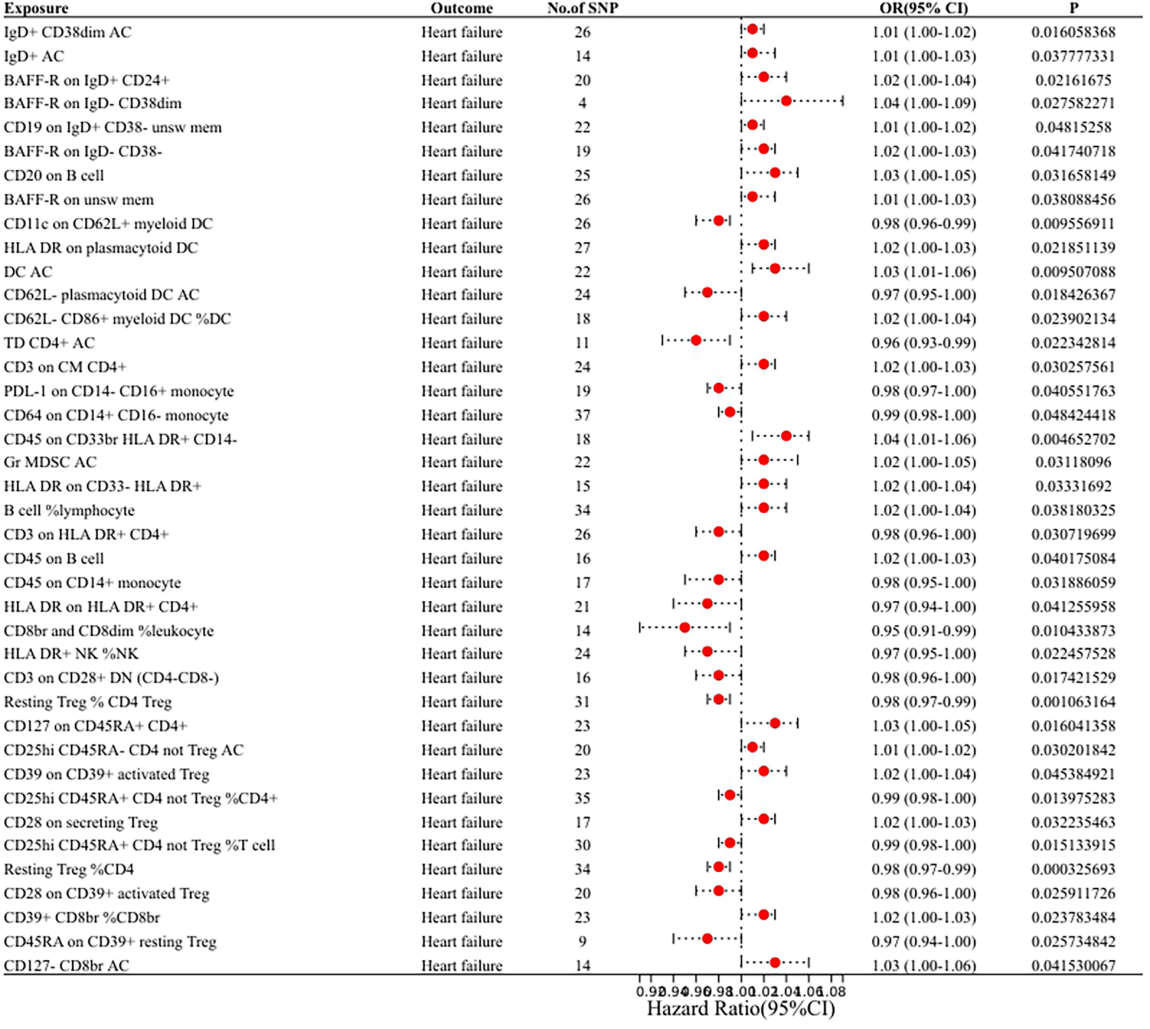
Forest plot of the MR analysis results.

In our research aimed at exploring the causal connection between immunophenotype traits and HF, we have implemented a two-sample MR analysis, with the IVW method being our key analytical strategy. At a significance of 0.001, we identified a significant causal relationship between 40 immunophenotypes and HF, of which 8 were in B cells, 5 in cDC, 2 in T cell maturation stages, 2 in monocytes, 3 in myeloid, 7 in TBNK and 13 in Treg. Detailed findings are presented in Table 1 and Figure 2.

**Table 1:** Summary of the GWAS included in this two sample mendelian randomization study.

Panel	Immune cells	Outcome	Nsnp	Methods	Beta	OR (95%CI)	*P*-value	Heterogeneity		Horizontal pleiotrop	
								Cochran's Q	*P*-value	Egger intercept P	Global _P
B cell	IgD+ CD38dim Activated B cells	Heart failure	26	Inverse variance weighted	0.013001392	1.01 (1.00–1.02)	0.016058368	37.61166154	0.050456059	0.070493332	0.113
B cell	IgD+ Activated B cells	Heart failure	14	Inverse variance weighted	0.013741615	1.01 (1.00–1.03)	0.037777331	12.79516365	0.463755513	0.312269213	0.584666667
B cell	BAFF-R on IgD+ CD24+	Heart failure	20	Inverse variance weighted	0.019508785	1.02 (1.00–1.04)	0.02161675	16.37832216	0.631905881	0.651833204	0.678333333
B cell	BAFF-R on IgD− CD38dim	Heart failure	4	Inverse variance weighted	0.043310779	1.04 (1.00–1.09)	0.027582271	2.22527228	0.52698769	0.950871813	0.722333333
B cell	CD19 on IgD+ CD38− unsw mem	Heart failure	22	Inverse variance weighted	0.011687977	1.01 (1.00–1.02)	0.04815258	16.06547356	0.765961162	0.959746797	0.808666667
B cell	BAFF-R on IgD− CD38−	Heart failure	19	Inverse variance weighted	0.016848657	1.02 (1.00–1.03)	0.041740718	13.27016684	0.775279638	0.805844502	0.816
B cell	CD20 on B cell	Heart failure	25	Inverse variance weighted	0.025171002	1.03 (1.00–1.05)	0.031658149	17.48367635	0.827313672	0.544190594	0.847333333
B cell	BAFF-R on unsw mem	Heart failure	26	Inverse variance weighted	0.014275199	1.01 (1.00–1.03)	0.038088456	14.66810871	0.948800084	0.133533746	0.953333333
cDC	CD11c on CD62l+ myeloid DC	Heart failure	26	Inverse variance weighted	−0.021073351	0.98 (0.96–0.99)	0.009556911	31.95073734	0.159442514	0.884137329	0.238666667
cDC	HLA DR on plasmacytoid DC	Heart failure	27	Inverse variance weighted	0.015261794	1.02 (1.00–1.03)	0.021851139	29.3953645	0.293412149	0.805330896	0.249
cDC	Activated DC	Heart failure	22	Inverse variance weighted	0.033511779	1.03 (1.01–1.06)	0.009507088	25.73729968	0.216643362	0.297763654	0.274
cDC	CD62l− plasmacytoid DC AC	Heart failure	24	Inverse variance weighted	−0.029171703	0.97 (0.95–1.00)	0.018426367	23.61160199	0.425569022	0.380476655	0.461333333
cDC	CD62l− CD86+ myeloid DC%DC	Heart failure	18	Inverse variance weighted	0.023347406	1.02 (1.00–1.04)	0.023902134	15.30948161	0.573200737	0.294846927	0.659666667
Maturation stages of T cell	TD CD4+ Activated T cells	Heart failure	11	Inverse variance weighted	−0.041283845	0.96 (0.93–0.99)	0.022342814	14.527626	0.150256156	0.385555852	0.185333333
Maturation stages of T cell	CD3 on CM CD4+	Heart failure	24	Inverse variance weighted	0.017652811	1.02 (1.00–1.03)	0.030257561	19.5716211	0.667595191	0.596816037	0.645666667
Monocyte	PDL-1 on CD14− CD16+monocyte	Heart failure	19	Inverse variance weighted	−0.016755738	0.98 (0.97–1.00)	0.040551763	25.21652038	0.119067367	0.174029571	0.152333333
Monocyte	CD64 on CD14+ CD16− monocyte	Heart failure	37	Inverse variance weighted	−0.008007007	0.99 (0.98–1.00)	0.048424418	28.68598567	0.801873514	0.330699479	0.842
Myeloid cell	CD45 on CD33br HLA DR+ CD14−	Heart failure	18	Inverse variance weighted	0.036290042	1.04 (1.01–1.06)	0.004652702	24.03157286	0.118579647	0.514015209	0.136666667
Myeloid cell	Gr MDSC Activated	Heart failure	22	Inverse variance weighted	0.024172629	1.02 (1.00–1.05)	0.03118096	23.04680223	0.341488084	0.691170035	0.348333333
Myeloid cell	HLA DR on CD33− HLA DR+	Heart failure	15	Inverse variance weighted	0.018043622	1.02 (1.00–1.04)	0.03331692	13.15842076	0.514089099	0.657538671	0.448666667
TBNK	B cell%lymphocyte	Heart failure	34	Inverse variance weighted	0.022148488	1.02 (1.00–1.04)	0.038180325	45.99067889	0.065905612	0.4750612	0.093666667
TBNK	CD3 on HLA DR+ CD4+	Heart failure	26	Inverse variance weighted	−0.023522317	0.98 (0.96–1.00)	0.030719699	25.47053896	0.436266803	0.640272338	0.493333333
TBNK	CD45 on B cell	Heart failure	16	Inverse variance weighted	0.015340934	1.02 (1.00–1.03)	0.040175084	15.47802658	0.41756316	0.151523743	0.498
TBNK	CD45 on CD14+ monocyte	Heart failure	17	Inverse variance weighted	−0.024602623	0.98 (0.95–1.00)	0.031886059	12.62073568	0.700259589	0.769277534	0.756666667
TBNK	HLA DR on HLA DR+ CD4+	Heart failure	21	Inverse variance weighted	−0.029043312	0.97 (0.94–1.00)	0.041255958	12.97009585	0.87866341	0.446003133	0.868
TBNK	CD8br and CD8dim%leukocyte	Heart failure	14	Inverse variance weighted	−0.054621721	0.95 (0.91–0.99)	0.010433873	6.821848943	0.911097352	0.396528899	0.925
TBNK	HLA DR+NK%NK	Heart failure	24	Inverse variance weighted	−0.026276408	0.97 (0.95–1.00)	0.022457528	13.75733288	0.933610147	0.568749677	0.95
Treg	CD3 on CD28+ DN (CD4-CD8-)	Heart failure	16	Inverse variance weighted	−0.025083003	0.98 (0.96–1.00)	0.017421529	23.66874303	0.070936584	0.438664803	0.087
Treg	Resting Treg% CD4 Treg	Heart failure	31	Inverse variance weighted	−0.017267764	0.98 (0.97–0.99)	0.001063164	40.86827589	0.089112917	0.474998502	0.124666667
Treg	CD127 on CD45RA+ CD4+	Heart failure	23	Inverse variance weighted	0.02580831	1.03 (1.00–1.05)	0.016041358	29.49558028	0.131248423	0.885772434	0.143
Treg	CD25hi CD45RA− CD4 not Treg AC	Heart failure	20	Inverse variance weighted	0.012592394	1.01 (1.00–1.02)	0.030201842	25.05037863	0.158890618	0.226265036	0.227333333
Treg	CD39 on CD39+ activated Treg	Heart failure	23	Inverse variance weighted	0.018135528	1.02 (1.00–1.04)	0.045384921	27.66621138	0.187008529	0.403826678	0.282
Treg	CD25hi CD45RA+ CD4 not Treg%CD4+	Heart failure	35	Inverse variance weighted	−0.013849485	0.99 (0.98–1.00)	0.013975283	39.59843915	0.234296975	0.382139921	0.285
Treg	CD28 on secreting Treg	Heart failure	17	Inverse variance weighted	0.016193936	1.02 (1.00–1.03)	0.032235463	20.08110022	0.216590121	0.954012376	0.296
Treg	CD25hi CD45RA+ CD4 not Treg%T cell	Heart failure	30	Inverse variance weighted	−0.01357461	0.99 (0.98–1.00)	0.015133915	32.7223325	0.289079322	0.57044318	0.358333333
Treg	Resting Treg%CD4	Heart failure	34	Inverse variance weighted	−0.018514319	0.98 (0.97–0.99)	0.000325693	35.28321293	0.360722166	0.947426567	0.409666667
Treg	CD28 on CD39+ activated Treg	Heart failure	20	Inverse variance weighted	−0.019664707	0.98 (0.96–1.00)	0.025911726	19.62934798	0.41718369	0.671981348	0.426666667
Treg	CD39+ CD8br%CD8br	Heart failure	23	Inverse variance weighted	0.015778735	1.02 (1.00–1.03)	0.023783484	23.65515653	0.365561473	0.826804257	0.454
Treg	CD45RA on CD39+ resting Treg	Heart failure	9	Inverse variance weighted	−0.032220296	0.97 (0.94–1.00)	0.025734842	8.542046977	0.38238665	0.695750664	0.515666667
Treg	CD127− CD8br AC	Heart failure	14	Inverse variance weighted	0.029460172	1.03 (1.00–1.06)	0.041530067	8.160550335	0.832985591	0.570566361	0.853333333

#### 3.2.1 B cell

We found that IgD+ CD38dim Activated B cells [OR (95% CI): 1.01 (1.00–1.02)], IgD+ Activated B cells [OR (95% CI): 1.01 (1.00–1.03)], BAFF-R on IgD+ CD24+ [OR (95% CI): 1.02 (1.00–1.04)], BAFF-R on IgD− CD38dim [OR (95% CI): 1.04 (1.00–1.09)], CD19 on IgD+ CD38− unsw mem [OR (95% CI): 1.01 (1.00–1.02)], BAFF-R on IgD− CD38− [OR (95% CI): 1.02 (1.00–1.03)], CD20 on B cell [OR (95% CI): 1.03 (1.00–1.05)], BAFF-R on unsw mem [OR (95% CI): 1.01 (1.00–1.03)] were risk factors for HF.

#### 3.2.2 cDC

We found that HLA DR on plasmacytoid DC [OR (95% CI): 1.02 (1.00–1.03)], Activated DC [OR (95% CI): 1.03 (1.01–1.06)], CD62l− CD86 +myeloid DC%DC Activated [OR (95% CI): 1.02 (1.00–1.04)] were risk factors for HF. CD11c on CD62l+ myeloid DC [OR (95% CI): 0.98 (0.96–0.99)], CD62l− plasmacytoid DC (OR (95% CI): 0.97 (0.95–1.00) were protective against HF.

#### 3.2.3 Maturation stages of T cell

We found that CD3 on CM CD4+ [OR (95% CI): 1.02 (1.00–1.03)] was a risk factor for HF.TD CD4+ Activated T cells [OR (95% CI): 0.96 (0.93–0.99)] was protective against HF.

#### 3.2.4 Monocyte

We found that PDL-1 on CD14− CD16+ monocyte [OR (95% CI): 0.98 (0.97–1.00)], CD64 on CD14+ CD16− monocyte [OR (95% CI): 0.99 (0.98–1.00)] were protective against HF.

#### 3.2.5 Myeloid

We found that CD64 on CD14+ CD16− monocyte [OR (95% CI): 1.04 (1.01–1.06)], Gr MDSC Activated [OR (95% CI): 1.02 (1.00–1.05)], HLA DR on CD33− HLA DR+ [OR (95% CI): 1.02 (1.00–1.04)] were risk factors for HF.

#### 3.2.6 TBNK

We found that B cell%lymphocyte [OR (95% CI): 1.02 (1.00–1.04)], CD45 on B cell [OR (95% CI): 1.02 (1.00–1.03)] were risk factors for HF. CD3 on HLA DR+ CD4+ [OR (95% CI): 0.98 (0.96–1.00)], CD45 on B cells [OR (95% CI): 0.98 (0.96–1.00)], CD45 on CD14+ monocytes [OR (95% CI): 0.98 (0.95–1.00)], HLA DR on HLA DR+ CD4+ [OR (95% CI): 0. 97 (0.94–1.00)], CD8br and CD8dim%leukocyte [OR (95% CI): 0.95 (0.91–0.99)], HLA DR+ NK%NK [OR (95% CI): 0.97 (0.95–1.00)] were protective against HF.

#### 3.2.7 Treg

We found that CD127 on CD45RA+ CD4+ [OR (95% CI): 1.03 (1.00–1.05)], CD25hi CD45RA- CD4 not Treg AC (CD25hi CD45RA- CD4 not Treg Activated) [OR (95% CI): 1.01 (1.00–1.02)], CD39 on CD39+ activated Treg [OR (95%CI): 1.02 (1.00–1.04)], CD28 on secreting Treg [OR (95% CI): 1.02 (1.00–1.03)], CD39+ CD8br%CD8br [OR (95% CI): 1.02 (1.00–1.03)], CD127- CD8br AC (CD127- CD8br Activated Cytotoxic T lymphocytes) [OR (95% CI): 1.03 (1.00–1.06)] were risk factors for HF. CD3 on CD28+ DN (CD4-CD8-) [OR (95% CI): 0.98 (0.96–1.00)], Resting Treg%CD4 Treg [OR (95% CI): 0.98 (0.97–0.99)], CD25hi CD45RA+ CD4 not Treg%CD4+ [OR (95% CI): 0.99 (0.98–1.00)], CD25hi CD45RA+ CD4 not Treg%T cell [OR (95% CI): 0. 99 (0.98–1.00)], resting Treg%CD4 [OR (95% CI): 0.98 (0.97–0.99)], CD28 on CD39+ activated Treg [OR (95% CI): 0.98 (0.96–1.00)], CD45RA on CD39+ resting Treg [OR (95% CI): 0.97 (0.94–1.00)] were protective against HF.

### 3.3 Sensitivity analysis

In the MR-Egger intercept test and MR-PRESSO global tests conducted, there was no indication of heterogeneity or cross-sectional pleiotropy concerning the relationship between immunophenotypes and HF. The detailed findings are presented in Supplementary Table S2. In addition, we confirmed the stability of the results of the MR analyses by the leave-one-out analysis. Even after removing each SNP associated with the immunophenotype and HF, the overall results of the MR analysis remained unchanged, demonstrating the reliability of our findings. The stability of the results is also shown by scatter plots and funnel plots (Supplementary Figure S2). Cochran's *Q*-test showed no significant terogeneity (*P* > 0.05) (Supplementary Table S2).

## 4 Discussion

In this study, we used widely publicly available genomic data to explore in depth the causal relationship between 731 immune cell traits and heart failure. By applying rigorous statistical analysis methods, we provide a more reliable causal interpretation of the link between immune cell traits and heart failure.

Damage to the myocardium releases self-antigens such as troponin, which activate and mature B cells. Activated B cells cause cardiomyocyte apoptosis by secreting antibodies (17). Anti-cardiac antibodies secreted by B cells can bind to target cells or form antigen-antibody complexes that activate the complement system, causing further myocardial damage. Each time the myocardium is injured, exposed cardiac autoantigens are released and the memory B cell response leads to a sustained inflammatory response. B cells play a crucial role in the inflammatory process. They continuously influence the immune response by presenting antigens and secreting cytokines and chemokines. B cells play a crucial role in the regulation of immune responses by activating and recruiting various innate and adaptive immune cells, including neutrophils, macrophages, fibroblasts, and T cells (18–21). Once B cells are activated, they have the capacity to trigger the differentiation of CD4T cells into the Th1 subtype. These Th1 cells, in their turn, can induce cardiac fibrosis, a process that may result in detrimental alterations to the heart's structure (22). adaptive.

A significant increase in the number of tumour necrosis factor-α (TNF-α)-secreting B cells has been observed in studies of patients with dilated cardiomyopathy. same At the same time, serum levels of type III procollagen in these patients also tended to be elevated. The findings indicate a potential involvement of B cells that secrete TNF-α in the development of myocardial fibrosis (23). CD20 is a specific transmembrane protein expressed on the surface of mature B cells that acts as an activator and regulator of B cells and is involved in their proliferation and differentiation. In heart failure, CD20-expressing B cells may be involved in the process of cardiac remodelling, which may be related to their secretion of cytokines and chemokines. Binding of rituximab (RTX) to CD20 on the B cell membrane promotes apoptosis and leads to B cell depletion (24). Studies have shown that rituximab (RTX) inhibits pressure overload-induced cardiac remodelling and dysfunction in mice. This effect was associated with a reduction in the expression of pro-inflammatory cytokines and the production of IgG mediated by Th2 cytokines produced by B cells (25). Relevant studies in the treatment of heart failure have demonstrated that the administration of rituximab, which targets the CD20 receptor on B cells, can ameliorate cardiac hypertrophy and enhance cardiac performance in a murine model subjected to transverse aortic constriction (25). This suggests that therapeutic strategies targeting CD20-expressing B cells may help improve the prognosis of patients with heart failure. We therefore speculate that increased CD20 expression on B cells or an increased percentage of CD20-expressing B cells increases the risk of heart failure, which may be related to the activation state of B cells and cardiac remodelling. All eight B cells identified in our study are risk factors for HF. However, the specific role of B-cell subsets in heart failure is currently unknown (26, 27). Further studies are needed to validate the role of B cell subsets and to develop new targets for the treatment of HF.

Dendritic cells (DCs) are key antigen-presenting cells in the immune system, and their main functions include presenting antigens to T lymphocytes, secreting a variety of cytokines and growth factors, and playing a regulatory role in immune responses and inflammatory processes. In the heart, conventional dendritic cells (cDCs) exhibit a unique set of developmental trajectories, functional properties and phenotypic characteristics (28). Our study found that 3 DCs were risk factors for HF and 2 DCs were protective against HF. It was shown that the number of circulating cDCs was reduced in both ischaemic and non-ischaemic heart failure patients. Furthermore, the count of DCs is positively associated with the ejection fraction of the left ventricle (LVEF), and it exhibits an inverse correlation with the end-diastolic diameter of the left ventricle (LVEDd) (29). Depletion of cDCs in myocardial infarction has been shown to improve cardiac function after myocardial infarction, significantly improving left ventricular ejection fraction while reducing infarct size and ameliorating adverse cardiomyocyte hypertrophy (30). In a population of patients with decompensated HF, the study found a significant reduction in the number of circulating cDCs. After treatment, there was a correlation between a decrease in B-type natriuretic peptide (BNP) and troponin T levels and an increase in LVEF as the number of cDCs increased (31). However, after myocardial ischaemic injury, activation of cytotoxic CD8T cells via cross-initiating DCs mediates a sustained autoimmune response against the heart, leading to myocardial injury and impaired cardiac function (32, 33). Studies have shown that DCs play an important role in promoting fibrosis and that there is a direct quantitative relationship between the number of DCs and the degree of reparative fibrosis in infarcted myocardium (30, 34).

Monocytes display a high degree of heterogeneity, encompassing various subpopulations characterized by unique cell surface markers and distinct functional properties. These subpopulations have different characteristics in the inflammatory and fibrotic responses (35). After myocardial damage, conditions such as hypoxia and tissue ischemia offer a range of stimuli that activate monocytes, including the triggering of CD14, increased expression of Toll-like receptor 4 (TLR4) and production of C-reactive protein (CRP). Once activated, monocytes migrate towards the myocardial tissue, adhere to the vascular endothelium and further infiltrate into the myocardium to participate in the inflammatory and repair processes following injury (1). We found a protective effect of 2 monocytes against heart failure, which may be related to the antifibrotic effect of monocytes. Subpopulations of monocytes and macrophages can regulate fibrosis by secreting proteases to degrade the extracellular matrix and by secreting inflammatory markers that negatively regulate fibroblast function (CCL12, IFNγ) (1, 36–38). Macrophage subpopulations with anti-inflammatory properties may have an indirect antifibrotic effect by inhibiting fibroblast activation (35, 39).

Regulatory T cells (Tregs), a specific class of CD4(+) CD25(+) Foxp3(+) T cell subsets, play a key role. Their main functions are to limit the immune system's overreaction, to ensure the stability of the immune system and to improve immune tolerance in the peripheral region. Through these functions, Tregs help to prevent the immune system from attacking its own tissues (40). 6 Tregs were found to be risk factors for HF. 7 Tregs were protective against HF. Circulating Tregs in patients with chronic heart failure have a significantly lower frequency, impaired function and reduced Foxp3 expression, and this phenomenon is not related to the aetiology of heart failure (41). The beneficial effect of a lack of Ccl17 on mitigating myocardial inflammation and preventing negative changes in the structure of the left ventricle is mediated through the modulating influence of regulatory T cells, or Tregs (42). In addition to their anti-inflammatory effects, Tregs have the ability to stimulate cardiomyocyte regeneration and promote the restoration of vascular structure. Additionally, this regulatory function helps in preserving the equilibrium of the surrounding tissue environment (43). Tregs promote the repair of cardiac scar tissue and the regeneration of cardiomyocytes in the treatment of myocardial infarction. They do this by secreting specific acidic and cysteine-rich proteins that help to improve the microenvironment of the infarcted area while promoting collagen synthesis and maturation, thus supporting the reconstruction and stabilisation of the vascular structure (44). Tregs play a key role in the repair process after cardiac injury and can significantly reduce the programmed death of cardiomyocytes, which has a positive impact on the recovery of cardiac function (45). Furthermore, Tregs stimulate the division of heart muscle cells, via a paracrine signaling process (46). Notably, approximately one week after myocardial infarction, the researchers observed a specific subpopulation of Treg cells expressing TNF-α receptor 1, which showed impaired function and may have pro-inflammatory effects (47). This suggests that Tregs have different properties at different times after cardiac injury. Tregs are also involved in heart failure and heart failure-associated diseases through direct interaction with immune cells and parenchymal cells (48–55). Treatment with Tregs can sometimes lead to systemic immune disorders due to the complex association between Tregs and immune or parenchymal cells (56).

Although many studies have suggested that T cells are important players in inflammation after heart injury, our study identified only 2 types of T cells that are associated with heart failure and have opposite effects on heart failure (3, 57–61). In non-ischemic heart failure, T-cell activation is followed by the release of cytokines that induce cardiac fibrosis and hypertrophy (58). In the mouse transverse aortic constriction (TAC) model, mice lacking the CD4(+) T-cell subset (MHCIIKO) did not develop cardiac dilatation and showed less fibrosis, collagen accumulation and cross-linking compared to mice lacking the CD8(+) T-cell subset (CD8KO) (62). The upregulation of T-cell immunoglobulin and mucin domain-containing protein 3 (TIM-3) in individuals with chronic heart failure implies that TIM-3 could be involved in the impairment of T-cell function (63). In addition, injection of myocardial infarction-induced splenic CD4(+) AT2R(+) T cells into recipient rats with myocardial infarction has been shown to reduce infarct size and improve cardiac function (64).

## 5 Conclusion

In conclusion, our study suggests that there appears to be a causal effect between multiple immune cells on heart failure. Furthermore, this study highlights the intricate nature of immune cells involved in the development of HF. This discovery provides a new avenue for the development of therapeutic treatments for myocardial infarction and a new target for drug development.

## Data Availability

The original contributions presented in the study are included in the article/Supplementary Material, further inquiries can be directed to the corresponding authors.

## Supplementary material

Supplementary Figure S1 Leave-one-out analysis for the impact of individual SNPs on the association between immune cells and HF risk.

Supplementary Figure S2 The scatter plots and Scatterplots of associations between immune cells and HF risk.

Supplementary Table S1 Instrumental variables (selected SNPs) used in MR analysis of the association between HF and immune cells.

## References

[1] Wrigley BJ Lip GY Shantsila E. The role of monocytes and inflammation in the pathophysiology of heart failure. Eur J Heart Fail. (2011) 13:1161–71. 10.1093/eurjhf/hfr122 21952932

[2] Savarese G Becher PM Lund LH Seferovic P Rosano GMC Coats AJS. Global burden of heart failure: a comprehensive and updated review of epidemiology. Cardiovasc Res. (2023) 118:3272–87. 10.1093/cvr/cvac013 35150240

[3] Chiurchiù V Leuti A Saracini S Fontana D Finamore P Giua R Resolution of inflammation is altered in chronic heart failure and entails a dysfunctional responsiveness of T lymphocytes. FASEB J. (2019) 33:909–16. 10.1096/fj.201801017R 30052486

[4] Zhang Y Bauersachs J Langer HF. Immune mechanisms in heart failure. Eur J Heart Fail. (2017) 19:1379–89. 10.1002/ejhf.942 28891154

[5] Dick SA Epelman S. Chronic heart failure and inflammation: what do we really know? Circ Res. (2016) 119:159–76. 10.1161/CIRCRESAHA.116.308030 27340274

[6] Bacmeister L Schwarzl M Warnke S Stoffers B Blankenberg S Westermann D Inflammation and fibrosis in murine models of heart failure. Basic Res Cardiol. (2019) 114:19. 10.1007/s00395-019-0722-5 30887214

[7] Damås JK Gullestad L Aass H Simonsen S Fjeld JG Wikeby L Enhanced gene expression of chemokines and their corresponding receptors in mononuclear blood cells in chronic heart failure–modulatory effect of intravenous immunoglobulin. J Am Coll Cardiol. (2001) 38:187–93. 10.1016/S0735-1097(01)01335-3 11451272

[8] Iborra-Egea O Gálvez-Montón C Roura S Perea-Gil I Prat-Vidal C Soler-Botija C Mechanisms of action of sacubitril/valsartan on cardiac remodeling: a systems biology approach. NPJ Syst Biol Appl. (2017) 3:12. 10.1038/s41540-017-0013-4 28649439 PMC5460292

[9] Hu YL Wang K Chen YH Jin YL Guo Q Tang H. Causal relationship between immune cell phenotypes and risk of biliary tract cancer: evidence from Mendelian randomization analysis. Front Immunol. (2024) 15:1430551. 10.3389/fimmu.2024.1430551 39050844 PMC11266158

[10] Wang S Wang K Chen X Lin S. The relationship between autoimmune thyroid disease, thyroid nodules and sleep traits: a Mendelian randomization study. Front Endocrinol (Lausanne). (2022) 14:1325538. 10.3389/fendo.2023.1325538 PMC10982365 38562570

[11] Wang K Wang SJ Qin XZ Chen YF Chen YH Wang JW The causal relationship between gut microbiota and biliary tract cancer: comprehensive bidirectional Mendelian randomization analysis. Front Cell Infect Microbiol. (2024) 14:1308742. 10.3389/fcimb.2024.1308742 38558852 PMC10978781

[12] Chen YH Yang L Wang K An Y Wang YP Zheng YA Relationship between fatty acid intake and aging: a Mendelian randomization study. Aging. (2024) 16:5711–39. 10.18632/aging.205674 38535988 PMC11006485

[13] Wang K Wang SJ Chen YH Lu XC Wang DS Zhang Y Causal relationship between gut microbiota and risk of gastroesophageal reflux disease: a genetic correlation and bidirectional Mendelian randomization study. Front Immunol. (2024) 15:1327503. 10.3389/fimmu.2024.1327503 38449873 PMC10914956

[14] Wang S Wang K Chen X Chen D Lin S. Autoimmune thyroid disease and myasthenia gravis: a study bidirectional Mendelian randomization. Front Endocrinol (Lausanne). (2024) 15:1310083. 10.3389/fendo.2024.1310083 38405140 PMC10884276

[15] Wang K Wang JW Chen YH Long H Pan W Liu YF Causal relationship between gut microbiota and risk of esophageal cancer: evidence from Mendelian randomization study. Aging. (2024) 16:3596–611. 10.18632/aging.205547 38364235 PMC10929825

[16] Wang K Qin XZ Ran TJ Pan YD Hong Y Wang JW Causal link between gut microbiota and four types of pancreatitis: a genetic association and bidirectional Mendelian randomization study. Front Microbiol. (2023) 14:1290202. 10.3389/fmicb.2023.1290202 38075894 PMC10702359

[17] Staudt Y Mobini R Fu M Felix SB Kühn JP Staudt A. Beta1-adrenoceptor antibodies induce apoptosis in adult isolated cardiomyocytes. Eur J Pharmacol. (2003) 466:1–6. 10.1016/S0014-2999(03)01431-6 12679135

[18] Youker KA Assad-Kottner C Cordero-Reyes AM Trevino AR Flores-Arredondo JH Barrios R High proportion of patients with end-stage heart failure regardless of aetiology demonstrates anti-cardiac antibody deposition in failing myocardium: humoral activation, a potential contributor of disease progression. Eur Heart J. (2014) 35:1061–8. 10.1093/eurheartj/eht506 24375073

[19] van den Hoogen P de Jager SCA Huibers MMH Schoneveld AH Puspitasari YM Valstar GB Increased circulating IgG levels, myocardial immune cells and IgG deposits support a role for an immune response in pre- and end-stage heart failure. J Cell Mol Med. (2019) 23:7505–16. 10.1111/jcmm.14619 31557411 PMC6815814

[20] Latif N Baker CS Dunn MJ Rose ML Brady P Yacoub MH. Frequency and specificity of antiheart antibodies in patients with dilated cardiomyopathy detected using SDS-PAGE and western blotting. J Am Coll Cardiol. (1993) 22:1378–84. 10.1016/0735-1097(93)90546-D 7901255

[21] García-Rivas G Castillo EC Gonzalez-Gil AM Maravillas-Montero JL Brunck M Torres-Quintanilla A The role of B cells in heart failure and implications for future immunomodulatory treatment strategies. ESC Heart Fail. (2020) 7:1387–99. 10.1002/ehf2.12744 32533765 PMC7373901

[22] Sánchez-Trujillo L Vázquez-Garza E Castillo EC García-Rivas G Torre-Amione G. Role of adaptive immunity in the development and progression of heart failure: new evidence. Arch Med Res. (2017) 48:1–11. 10.1016/j.arcmed.2016.12.008 28577862

[23] Yu M Wen S Wang M Liang W Li H-H Long Q TNF-α-secreting B cells contribute to myocardial fibrosis in dilated cardiomyopathy. J Clin Immunol. (2013) 33:1002–8. 10.1007/s10875-013-9889-y 23558825

[24] Sánchez-Trujillo L Jerjes-Sanchez C Rodriguez D Panneflek J Ortiz-Ledesma C Garcia-Rivas G Phase II clinical trial testing the safety of a humanised monoclonal antibody anti-CD20 in patients with heart failure with reduced ejection fraction, ICFEr-RITU2: study protocol. BMJ Open. (2019) 9:e022826. 10.1136/bmjopen-2018-022826 PMC6475246 30918029

[25] Ma X-L Lin Q-Y Wang L Xie X Zhang Y-L Li H-H. Rituximab prevents and reverses cardiac remodeling by depressing B cell function in mice. Biomed Pharmacother. (2019) 114:108804. 10.1016/j.biopha.2019.108804 30909146

[26] Sun HR Kong XJ Wei KM Hao J Xi Y Meng LW Risk prediction model construction for post myocardial infarction heart failure by blood immune B cells. Front Immunol. (2023) 14:1163350. 10.3389/fimmu.2023.1163350 37287974 PMC10242647

[27] Adamo L Staloch LJ Rocha-Resende C Matkovich SJ Jiang WL Bajpai G Modulation of subsets of cardiac B lymphocytes improves cardiac function after acute injury. JCI Insight. (2018) 3(11):e120137. 10.1172/jci.insight.120137 29875326 PMC6124442

[28] Murphy TL Grajales-Reyes GE Wu X Tussiwand R Briseño CG Iwata A Transcriptional control of dendritic cell development. Annu Rev Immunol. (2016) 34:93–119. 10.1146/annurev-immunol-032713-120204 26735697 PMC5135011

[29] Pistulli R Hammer N Rohm I Kretzschmar D Jung C Figulla H-R Decrease of circulating myeloid dendritic cells in patients with chronic heart failure. Acta Cardiol. (2016) 71:165–72. 10.1080/AC.71.2.3141846 27090038

[30] Lee JS Jeong S-J Kim S Chalifour L Yun TJ Miah MA Conventional dendritic cells impair recovery after myocardial infarction. J Immunol. (2018) 201:1784–98. 10.4049/jimmunol.1800322 30097529

[31] Sugi Y Yasukawa H Kai H Fukui D Futamata N Mawatari K Reduction and activation of circulating dendritic cells in patients with decompensated heart failure. Int J Cardiol. (2011) 147:258–64. 10.1016/j.ijcard.2009.09.524 19923020

[32] Forte E Perkins B Sintou A Kalkat HS Papanikolaou A Jenkins C. Cross-priming dendritic cells exacerbate immunopathology after ischemic tissue damage in the heart. Circulation. (2021) 143:821–36. 10.1161/CIRCULATIONAHA.120.044581 33297741 PMC7899721

[33] Naito K Anzai T Sugano Y Maekawa Y Kohno T Yoshikawa T Differential effects of GM-CSF and G-CSF on infiltration of dendritic cells during early left ventricular remodeling after myocardial infarction. J Immunol. (2008) 181:5691–701. 10.4049/jimmunol.181.8.5691 18832728

[34] Nagai T Honda S Sugano Y Matsuyama T-A Ohta-Ogo K Asaumi Y Decreased myocardial dendritic cells is associated with impaired reparative fibrosis and development of cardiac rupture after myocardial infarction in humans. J Am Heart Assoc. (2014) 3:e000839. 10.1161/JAHA.114.000839 24895162 PMC4309075

[35] Kong P Christia P Frangogiannis NG. The pathogenesis of cardiac fibrosis. Cell Mol Life Sci. (2014) 71:549–74. 10.1007/s00018-013-1349-6 23649149 PMC3769482

[36] Nahrendorf M Swirski FK. Monocyte and macrophage heterogeneity in the heart. Circ Res. (2013) 112:1624–33. 10.1161/CIRCRESAHA.113.300890 23743228 PMC3753681

[37] DeLeon-Pennell KY Iyer RP Ero OK Cates CA Flynn ER Cannon PL Periodontal-induced chronic inflammation triggers macrophage secretion of Ccl12 to inhibit fibroblast-mediated cardiac wound healing. JCI Insight. (2018) 3(11):e120137. 10.1172/jci.insight.94207 28931761 PMC5621894

[38] Lee J-W Oh JE Rhee K-J Yoo B-S Eom YW Park SW Co-treatment with interferon-*γ* and 1-methyl tryptophan ameliorates cardiac fibrosis through cardiac myofibroblasts apoptosis. Mol Cell Biochem. (2019) 458:197–205. 10.1007/s11010-019-03542-7 31006829 PMC6616223

[39] Frangogiannis NG. Regulation of the inflammatory response in cardiac repair. Circ Res. (2012) 110:159–73. 10.1161/CIRCRESAHA.111.243162 22223212 PMC3690135

[40] Hu W Li J Cheng X. Regulatory T cells and cardiovascular diseases. Chin Med J (Engl). (2023) 136:2812–23. 10.1097/CM9.0000000000002875 37840195 PMC10686601

[41] Tang T-T Ding Y-J Liao Y-H Yu X Xiao H Xie J-J Defective circulating CD4CD25+ Foxp3+ CD127(low) regulatory T-cells in patients with chronic heart failure. Cell Physiol Biochem. (2010) 25:451–8. 10.1159/000303050 20332626

[42] Feng G Bajpai G Ma P Koenig A Bredemeyer A Lokshina I CCL17 aggravates myocardial injury by suppressing recruitment of regulatory T cells. Circulation. (2022) 145:765–82. 10.1161/CIRCULATIONAHA.121.055888 35113652 PMC8957788

[43] Yang J Liao X Yu J Zhou P. Role of CD73 in disease: promising prognostic indicator and therapeutic target. Curr Med Chem. (2018) 25:2260–71. 10.2174/0929867325666180117101114 29345574

[44] Xia N Lu YZ Gu MY Li NN Liu ML Jiao JJ A unique population of regulatory T cells in heart potentiates cardiac protection from myocardial infarction. Circulation. (2020) 142:1956–73. 10.1161/CIRCULATIONAHA.120.046789 32985264

[45] Zeng Z Yu K Chen L Li W Xiao H Huang Z. Interleukin-2/Anti-Interleukin-2 immune Complex attenuates cardiac remodeling after myocardial infarction through expansion of regulatory T cells. J Immunol Res. (2016) 2016:8493767. 10.1155/2016/8493767 27144181 PMC4837274

[46] Li JT Yang KY Tam RCY Chan VW Lan HY Hori S Regulatory T-cells regulate neonatal heart regeneration by potentiating cardiomyocyte proliferation in a paracrine manner. Theranostics. (2019) 9:4324–41. 10.7150/thno.32734 31285764 PMC6599663

[47] Bansal SS Ismahil MA Goel M Zhou G Rokosh G Hamid T Dysfunctional and proinflammatory regulatory T-lymphocytes are essential for adverse cardiac remodeling in ischemic cardiomyopathy. Circulation. (2019) 139:206–21. 10.1161/CIRCULATIONAHA.118.036065 30586716 PMC6322956

[48] Cheng X Yu X Ding Y-J Fu Q-Q Xie J-J Tang T-T The Th17/treg imbalance in patients with acute coronary syndrome. Clin Immunol. (2008) 127:89–97. 10.1016/j.clim.2008.01.009 18294918

[49] Hu H Wu J Cao C Ma L. Exosomes derived from regulatory T cells ameliorate acute myocardial infarction by promoting macrophage M2 polarization. IUBMB Life. (2020) 72:2409–19. 10.1002/iub.2364 32842172

[50] Cederbom L Hall H Ivars F. CD4+ CD25+ regulatory T cells down-regulate co-stimulatory molecules on antigen-presenting cells. Eur J Immunol. (2000) 30:1538–43. 10.1002/1521-4141(200006)30:6<1538::AID-IMMU1538>3.0.CO;2-X 10898488

[51] Chattopadhyay G Shevach EM. Antigen-specific induced T regulatory cells impair dendritic cell function via an IL-10/MARCH1-dependent mechanism. J Immunol. (2013) 191:5875–84. 10.4049/jimmunol.1301693 24218453 PMC3858537

[52] Weirather J Hofmann UDW Beyersdorf N Ramos GC Vogel B Frey A Foxp3+ CD4+ T cells improve healing after myocardial infarction by modulating monocyte/macrophage differentiation. Circ Res. (2014) 115:55–67. 10.1161/CIRCRESAHA.115.303895 24786398

[53] Tang T-T Yuan J Zhu Z-F Zhang W-C Xiao H Xia N Regulatory T cells ameliorate cardiac remodeling after myocardial infarction. Basic Res Cardiol. (2012) 107:232. 10.1007/s00395-011-0232-6 22189560

[54] Lim HW Hillsamer P Banham AH Kim CH. Cutting edge: direct suppression of B cells by CD4+ CD25+ regulatory T cells. J Immunol. (2005) 175:4180–3. 10.4049/jimmunol.175.7.4180 16177055

[55] Zhu Z-F Tang T-T Dong W-Y Li Y-Y Xia N Zhang W-C Defective circulating CD4+ LAP+ regulatory T cells in patients with dilated cardiomyopathy. J Leukoc Biol. (2015) 97:797–805. 10.1189/jlb.5A1014-469RR 25722319

[56] Lu Y Xia N Cheng X. Regulatory T cells in chronic heart failure. Front Immunol. (2021) 12:732794. 10.3389/fimmu.2021.732794 34630414 PMC8493934

[57] Bradshaw AD DeLeon-Pennell KY. T-cell regulation of fibroblasts and cardiac fibrosis. Matrix Biol. (2020) 91-92:167–75. 10.1016/j.matbio.2020.04.001 32438054 PMC7434661

[58] Nevers T Salvador AM Grodecki-Pena A Knapp A Velázquez F Aronovitz M Left ventricular T-cell recruitment contributes to the pathogenesis of heart failure. Circ Heart Fail. (2015) 8:776–87. 10.1161/CIRCHEARTFAILURE.115.002225 26022677 PMC4512916

[59] Nevers T Salvador AM Velazquez F Ngwenyama N Carrillo-Salinas FJ Aronovitz M. Th1 effector T cells selectively orchestrate cardiac fibrosis in nonischemic heart failure. J Exp Med. (2017) 214:3311–29. 10.1084/jem.20161791 28970239 PMC5679176

[60] Jane-wit D Tuohy VK. Autoimmune cardiac-specific T cell responses in dilated cardiomyopathy. Int J Cardiol. (2006) 112:2–6. 10.1016/j.ijcard.2006.05.007 16859767

[61] Li N Bian HJ Zhang J Li XX Ji XP Zhang Y. The Th17/treg imbalance exists in patients with heart failure with normal ejection fraction and heart failure with reduced ejection fraction. Clin Chim Acta. (2010) 411:1963–8. 10.1016/j.cca.2010.08.013 20713031

[62] Laroumanie F Douin-Echinard V Pozzo J Lairez O Tortosa F Vinel C CD4+ T cells promote the transition from hypertrophy to heart failure during chronic pressure overload. Circulation. (2014) 129:2111–24. 10.1161/CIRCULATIONAHA.113.007101 24657994

[63] Yu HW Dong YY Dang YH. The modulatory activity of T cell immunoglobulin and mucin domain-containing protein 3 on T lymphocytes in patients with chronic heart failure. Zhonghua Yi Xue Za Zhi. (2020) 100:1315–9. 10.3760/cma.j.cn112137-20190823-01876 32375439

[64] Skorska A von Haehling S Ludwig M Lux CA Gaebel R Kleiner G The CD4(+) AT2R(+) T cell subpopulation improves post-infarction remodelling and restores cardiac function. J Cell Mol Med. (2015) 19:1975–85. 10.1111/jcmm.12574 25991381 PMC4549048

